# Inheritance Patterns, Dominance and Cross-Resistance of Cry1Ab- and Cry1Ac-Selected *Ostrinia furnacalis* (Guenée)

**DOI:** 10.3390/toxins6092694

**Published:** 2014-09-11

**Authors:** Tiantao Zhang, Mingxia He, Angharad M. R. Gatehouse, Zhenying Wang, Martin G. Edwards, Qing Li, Kanglai He

**Affiliations:** 1 The State Key Laboratory for Biology of Plant Diseases and Insect Pests, Institute of Plant Protection, Chinese Academy of Agricultural Sciences, Beijing 100193, China; E-Mails: ttzhang@ippcaas.cn (T.Z.); hemingxia2605@126.com (M.H.); zywang@ippcaas.cn (Z.W.); 2 Newcastle Institute for Research on Sustainability, School of Biology, University of Newcastle, Newcastle upon Tyne NE1 7RU, UK; E-Mails: a.m.r.gatehouse@newcastle.ac.uk (A.M.R.G.); martin.edwards@newcastle.ac.uk (M.G.E.); 3 Agronomy College, Sichuan Agricultural University, Chengdu 611130, China; E-Mail: liq8633@163.com

**Keywords:** *Ostrinia furnacalis*, Cry1 toxins, cross-resistance, resistance inheritance

## Abstract

Two colonies of Asian corn borer, *Ostrinia furnacalis* (Guenée), artificially selected from a Bt-susceptible colony (ACB-BtS) for resistance to Cry1Ab (ACB-AbR) and Cry1Ac (ACB-AcR) toxins, were used to analyze inheritance patterns of resistance to Cry1 toxins. ACB-AbR and ACB-AcR evolved significant levels of resistance, with resistance ratios (RR) of 39-fold and 78.8-fold to Cry1Ab and Cry1Ac, respectively. The susceptibility of ACB-AbR larvae to Cry1Ac and Cry1F toxins, which had not previously been exposed, were significantly reduced, being >113-fold and 48-fold, respectively. Similarly, susceptibility of ACB-AcR larvae to Cry1Ab and Cry1F were also significantly reduced (RR > nine-fold, RR > 18-fold, respectively), indicating cross-resistance among Cry1Ab, Cry1Ac, and Cry1F toxins. However, ACB-AbR and ACB-AcR larvae were equally susceptible to Cry1Ie as were ACB-BtS larvae, indicating no cross-resistance between Cry1Ie and Cry1Ab or Cry1Ac toxins; this may provide considerable benefits in preventing or delaying the evolution of resistance in ACB to Cry1Ab and Cry1Ac toxins. Backcrossing studies indicated that resistance to Cry1Ab toxin was polygenic in ACB-AbR, but monogenic in ACB-AcR, whilst resistance to Cry1Ac toxin was primarily monogenic in both ACB-AbR and ACB-AcR, but polygenic as resistance increased.

## 1. Introduction

*Bacillus thuringiensis* (Bt) is a valuable source of insecticidal proteins used in pest control, both in spray formulations and when expressed in genetically modified crops. Bt has been adopted as the most promising alternative to synthetic insecticides [1]. Bt transgenic crops have been highly successful and beneficial to the agricultural industry, leading to lower yield losses and reducing the use of chemical pesticides and fossil fuels. Despite these benefits, there remains the ever-present concern that insects will evolve resistance to the Bt toxins currently in use, seriously threatening the longevity of this technology. Since the global commercialization of transgenic Bt crops the total planted area has increased more than 100-fold from 1.7 million hectares in 1996 to 175 million hectares in 2013, thus, making biotech crops the fastest adopted crop technology in the history of modern agriculture [2].

Since the first report on insect resistance to Bt was published in 1985 [3], a number of resistance colonies have been selected under laboratory conditions or identified in field grown Bt crops. The use of Bt toxins is most widespread in the control of lepidopteran pests, therefore it is by no coincidence that Bt resistance has evolved in several field populations of *Plutella xylostella* [4,5], *Ostrinia nubilalis* (European Corn Borer) [6,7], and *Pectinophora gossypiella* (Pink bollworm) [8,9,10]. In addition, resistance has also been identified in greenhouse populations of *Trichoplusia ni* (Hübner) [11], while other economically important insect pests have demonstrated the potential to evolve high levels of resistance to Bt under laboratory selection conditions [3,12,13,14].

The Asian corn borer (ACB), *Ostrinia furnacalis* (Guenée) (Lepidoptera: Crambidae), is the most destructive corn stalk boring pest in Asia, particularly in China and the Philippines, with losses estimated to be six to nine million tons per year with significantly more losses in an outbreak year [15,16]. Earlier studies in China showed that Cry1Ab-expressing maize MON810 and Bt11 could effectively protect against the Asian corn borer and other lepidopteron pests [17,18,19,20]. However, the potential of resistance evolution has been documented in the laboratory resulting in Cry1Ab-resistant colonies of ACB with the propensity to survive well on two Bt maize events [21]. Under laboratory conditions and using artificial diets containing Cry1Ab protein, resistance of *O. furnacalis* towards the Bt toxin increased more than 100-fold after 35 generations [22]. Furthermore, experiments with Cry1Ac protein incorporated into artificial diet showed that the level of Bt resistance in *O. furnacalis* increased 14-fold after 27 generations of selection, and continued to increase, so after 82 generations the level of resistance reached 48.9-fold that of the original insect colony [23]. Similar studies in the US have shown that laboratory strains of *O. nubilalis* evolved resistance when selected on formulations of Dipel, Cry1Ac, Cry1Ab and Cry1F [24,25,26,27]; Bt maize has been available in the US since 1996, and these case studies show the potential for resistance. Furthermore, Devos *et al.* demonstrated that field-selected “populations” of the Coleopteran pest *Diabrotica virgifera virgifera* (Western corn rootworm, WCR) resistant to Cry3Bb1 caused unexpected levels of damage to Bt-maize MON88017 [28].

Understanding the mode of inheritance of Bt resistance is key to developing resistance management strategies and facilitating the selection and design of engineered bacterial strains, which can increase host range and durability to resistance. In this study, inheritance of Cry1Ab- and Cry1Ac- resistance in laboratory-selected *O. furnacalis* colonies was analysed. Mendelian crosses were carried out to determine maternal effect, sex linkage, and dominance, while backcrossing experiments were used to estimate the number of loci (one or multiple loci) involved in Cry1Ab- and Cry1Ac- resistance.

## 2. Results

### 2.1. Levels of Cross-Resistance

The LC_50_ values for the susceptible (ACB-BtS) and Cry1Ab-selected colonies (ACB-AbR) when exposed to Cry1Ab were significantly different (0.12 and 4.73 μg/g Cry1Ab toxin, respectively), with a resistance ratio of 39.42 (Table 1). LC_50_ values for both sets of F_1_ offspring produced from reciprocal crosses between ACB-AbR and ACB-BtS were intermediate to those of the parents, and were not significantly different from each other (Table 1).

**Table 1:** Susceptibility of Cry1Ab-selected (ACB-AbR, R) and susceptible (ACB-BtS, S) strains of *O. furnacalis* and their F_1_ progenies of reciprocal crosses to 4 Bt toxins.

Bt toxins	Colony	LC_50_ (95%FL) μg/g	Resistance ratio RR_50_	χ^2^	Slope ± SE
Cry1Ab	ACB-BtS	0.12 (0.05–0.21)	-	8.19	1.05 ± 0.11
	ACB-AbR	4.73 (2.67–8.07)	39.42	19.91	1.0 ± 0.06
	F_1_ (S×R)	1.40 (0.86–2.36)	11.67	6.78	0.85 ± 0.08
	F_1_ (R×S)	2.01 (1.25–3.34)	16.75	2.19	0.77 ± 0.07
Cry1Ac	ACB-BtS	0.10 (0.07–0.15)	-	8.44	1.05 ± 0.06
	ACB-AbR	11.34 (6.88–23.77)	113.40	3.34	0.86 ± 0.24
	F_1_ (S×R)	1.29 (0.88–1.89)	12.90	9.03	0.89 ± 0.08
	F_1_ (R×S)	1.82 (1.12–3.05)	18.20	5.52	0.82 ± 0.07
Cry1F	ACB-BtS	0.64 (0.46–1.05)	-	3.60	0.90 ± 0.09
	ACB-AbR	31.20 (17.98–114.40)	48.75	12.10	0.88 ± 0.19
	F_1_ (S×R)	1.99 (1.24–3.31)	3.11	5.78	0.81 ± 0.07
	F_1_ (R×S)	2.97 (1.87–4.95)	4.64	4.08	0.70 ± 0.07
Cry1Ie	ACB-BtS	1.36 (0.54–2.83)	-	15.77	1.80 ± 0.13
	ACB-AbR	1.95 (1.23–3.14)	1.60	12.20	1.08 ± 0.10
	F_1_ (S×R)	1.00 (0.58–1.76)	0.74	7.09	0.90 ± 0.08
	F_1_ (R×S)	1.10 (0.74–1.64)	0.81	3.50	0.87 ± 0.08

When exposed to Cry1Ac toxin, the LC_50_ values for ACB-BtS and ACB-AbR again were significantly different; but the resistance ratio was numerically higher (113.40) than when they were exposed to Cry1Ab toxin (Table 1), which indicated cross-resistance. The LC_50_ values for both sets of offspring produced from the reciprocal crosses were intermediate to the parents, and were not significantly different from each other (Table 1).

When exposed to Cry1F toxin, the LC_50_ values for ACB-BtS and ACB-AbR once again were significantly different, producing a resistance ratio of 48.75 (Table 1). This too indicates cross-resistance. The LC_50_ values for both sets of offspring produced from the reciprocal crosses were intermediate to the parents, and were not significantly different from each other (Table 1). However, when exposed to Cry1Ie toxin, none of the LC_50_ values produced from ACB-BtS and ACB-AbR nor the F_1_ offspring were significantly different from each other. Exposure of ACB-AbR larvae to Cry1Ie showed resistance ratios of 1.95 at the LC_50_ (Table 1); similarly, the LC_50_ of the F_1_ offspring from the reciprocal crosses were 1.00 and 1.10, indicating no cross-resistance to Cry1Ie.

By contrast, the LC_50_ values for ACB-BtS and the Cry1Ac-selected colony ACB-AcR were significantly different (0.10 and 7.88 μg/g Cry1Ab toxin, respectively). The resistance ratio was 78.8 (Table 2). LC_50_ values for both sets of F_1_ offspring produced from reciprocal crosses between ACB-AbR and BtS were intermediate to the parents, and were not significantly different from each other (Table 2).

**Table 2:** Susceptibility of ACB-AcR and ACB-BtS strains and their F_1_ progenies from reciprocal crosses to four Bt toxins.

Bt Toxins	Colony	LC_50_ (95% FL) μg/g	Resistance Ratio RR_50_	χ^2^	Slope ± SE
Cry1Ab	ACB-BtS	0.12 (0.05–0.21)	-	8.19	1.05 ± 0.11
	ACB-AcR	1.17 (0.65–1.96)	9.75	18.85	0.95 ± 0.06
	S×R	1.20 (0.82–1.74)	10.00	4.70	0.94 ± 0.08
	R×S	1.51 (1.03–2.23)	12.58	3.16	0.91 ± 0.08
Cry1Ac	ACB-BtS	0.10 (0.07–0.15)	-	8.44	1.05 ± 0.06
	ACB-AcR	7.88 (3.59–18.61)	78.80	28.94	0.79 ± 0.06
	S×R	1.28 (0.89–1.84)	12.80	4.89	1.99 ± 0.08
	R×S	2.63 (1.71–4.19)	26.30	3.11	0.76 ± 0.07
Cry1F	ACB-BtS	0.64 (0.46–1.05)	-	3.60	0.90 ± 0.09
	ACB-AcR	11.61 (6.70–23.28)	18.14	8.54	0.61 ± 0.05
	S×R	2.70 (1.57–5.09)	4.22	6.72	0.84 ± 0.08
	R×S	4.76 (2.45–10.70)	7.44	8.39	0.75 ± 0.07
Cry1Ie	ACB-BtS	1.36 (0.54–2.83)	-	15.77	1.8 ± 0.13
	ACB-AcR	1.88 (0.98–3.42)	1.38	23.60	0.96 ± 0.06
	S×R	1.34 (0.73–2.44)	0.99	9.80	0.98 ± 0.08
	R×S	1.73 (1.11–2.77)	1.27	5.96	0.92 ± 0.08

Cross-resistance to Cry1Ab within the ACB-AcR colony showed a resistance ratio of 9.75, which was much lower than the resistance ratio with colony ACB-AbR. The F_1_ offspring from the reciprocal crosses showed a slightly greater level of cross-resistance to Cry1Ab than their resistant parent, with resistance ratios of 10.00 and 12.58.

When challenged with Cry1F, the ACB-AcR colony revealed a cross-resistance level higher than that seen with Cry1Ab, with a resistance ratio of 18.14. In contrast, the F_1_ offspring of the reciprocal crosses to Cry1F showed a reduced level of cross-resistance in comparison to Cry1Ab, with cross-resistance ratios of 4.22 and 7.44 at the LC_50_.

The ACB-AcR colony responded to Cry1Ie with a similar trend to that seen with the ACB-AbR colony, showing no cross-resistance. Again the F_1_ offspring from the reciprocal crosses between the ACB-AcR colony gave no significant differences in cross-resistance from those seen with the susceptible and resistant parents upon exposure to Cry1Ie.

### 2.2. Estimation of Dominance

Dominance estimations at different concentrations of the Cry1 Bt toxin revealed that resistance of the F_1_ offspring was generally dominant at low treatment concentrations, but that dominance decreased as the treatment concentrations increased (Table 3). For example, for F_1_ offspring from reciprocal crosses between ACB-AbR and ACB-BtS, treatment with Cry1Ab showed complete dominance (1.00) at 0.1 µg/g, but when the concentration reached the maximum 10.00 µg/g, dominance declined to a semirecessive state. Similarly, with F_1_ offspring from reciprocal crosses between ACB-AcR and ACB-BtS, tests with Cry1Ab showed complete dominance (1.00) at 0.10 µg/g, but at a concentration of 10.00 µg/g, dominance shifted from dominant to semidominant.

**Table 3:** Estimation of dominance for F_1_ progenies of reciprocal crosses between ACB-AbR and ACB-BtS or ACB-AcR and ACB-BtS.

Toxin	Concentration (µg/g)	*h* value (Crosses)			
		ACB-AbRS	ACB-SAbR	ACB-AcRS	ACB-SAcR
Cry1Ab	0.10	1.00	1.00	1.00	1.00
	1.00	0.90	0.78	1.00	1.00
	10.00	0.44	0.35	0.80	0.74
Cry1Ac	0.10	1.00	1.00	1.00	1.00
	1.00	0.98	0.79	0.83	0.67
	10.00	0.52	0.20	0.43	0.20
Cry1F	0.10	1.00	1.00	1.00	1.00
	1.00	0.90	0.80	0.70	1.00
	10.00	0.60	0.40	0.40	0.50
	100.00	0.30	0.10	0.15	0.06

Thus colonies of ACB-AbR and ACB-AcR showed the same mode of resistance inheritance, both from complete dominance to semi-recessive as treatment concentration increased. Cross-resistance to Cry1F also ranged from dominant to semi-recessive as treatment concentration increased.

Both resistant colonies, and the offspring from the reciprocal crosses between ACB-AbR and ACB-AcR were also evaluated for their resistance to the different Cry1 toxins (Table 4). Exposure to Cry1Ab resulted in no significant differences between each of the hybrid crosses and the homozygous resistant parents; these results are synonymous with autosomal inheritance and with no maternal effects. However, when exposed to Cry1Ac and Cry1F, the hybrid offspring from both crosses were more resistant than the individual homozygous resistant parents, indicating resistance and cross-resistance alleles were strongly complementary. The resistance ratios were 0.82–1.60 to Cry1Ie in ACB-AbR, ACB-AcR and the hybrid offspring of the reciprocal crosses, indicating that cross-resistance was not observed.

**Table 4:** Susceptibility of ACB-AbR and ACB-AcR strains and their progenies of reciprocal crosses toward four Bt toxins.

Toxins	Colony	LC_50_ (95% FL) µg/g	Resistance ratio	χ^2^	Slope ± SE
Cry1Ab	ACB-AbR	4.73 (2.67–8.07)	39.42	19.91	1.00 ± 0.06
	ACB-AcR	1.17 (0.65–1.96)	9.75	18.85	0.95 ± 0.06
	ACB-AbcR	2.85 (1.96–4.01)	23.75	11.87	1.30 ± 0.08
	ACB-AcbR	1.55 (0.93–2.43)	12.92	18.80	1.24 ± 0.07
Cry1Ac	ACB-AbR	11.34 (6.88–23.77)	113.40	3.34	0.86 ± 0.24
	ACB-AcR	7.88 (3.59–18.61)	78.80	28.94	0.79 ± 0.06
	ACB-AbcR	10.25 (7.94–13.35)	102.50	5.14	0.88 ± 0.06
	ACB-AcbR	19.81 (15.41–25.87)	198.10	3.90	0.99 ± 0.08
Cry1F	ACB-AbR	31.20 (17.98–114.40)	48.75	12.10	0.88 ± 0.19
	ACB-AcR	11.61 (6.70–23.28)	18.14	8.54	0.61 ± 0.05
	ACB-AbcR	35.80 (22.00–67.61)	55.94	1.24	0.59 ± 0.06
	ACB-AcbR	47.78 (29.56–91.45)	74.66	4.13	0.66 ± 0.07
Cry1Ie	ACB-AbR	1.95 (1.23–3.14)	1.60	12.20	1.08 ± 0.10
	ACB-AcR	1.88 (0.98–3.42)	1.38	23.60	0.96 ± 0.06
	ACB-AbcR	1.66 (1.00–2.57)	1.22	16.69	1.16 ± 0.08
	ACB-AcbR	1.12 (0.61–1.94)	0.82	21.65	0.99 ± 0.06

### 2.3. Inheritance of Resistance

The offspring from the backcross ACB-AbRS×ACB-AbR and the F_1_ colony backcrossed to the original parental colony, were tested against Cry1Ab. All interactions were significant across the range of concentrations used (*P* < 0.05); the results also showed that the average difference between actual mortality and expected mortality was 17.20% (Table 5). These results are not significantly different from the monofactorial model, and, thus, indicated the inheritance of ACB-AbR to Cry1Ab does not fit the monogenic model but can be explained by the polygenic model. In contrast, the results for response of ACB-AcRS×ACB-BtS to Cry1Ab showed no significant difference to the monogenic model across the range of concentrations of 0.05–50.00 µg/g (*P* > 0.05), indicating that inheritance is monogenic.

**Table 5:** Indirect tests for deviation between observed and expected mortality for monogenic and additive polygenic models in ACB-AbR and ACB-AcR to Cry1Ab.

Strains		Concentration (µg/g)						
		0.05	0.25	0.50	2.50	5.00	25.00	50.00
ACB-AbRS×ACBAbR	Actual mortality (%)	18.06	27.08	41.67	70.14	78.47	93.75	97.22
	Expected mortality (%)	11.63	16.03	23.09	42.71	53.47	73.44	85.59
	χ^2^	5.78	13.06	27.98	44.28	36.17	30.46	15.80
	*P*	0.02	0.00	0.00	0.00	0.00	0.00	0.00
	Mean difference (%)	17.20						
ACB-AcRS×ACB-BtS	Actual mortality (%)	21.53	40.97	48.61	79.17	86.11	95.14	97.92
	Expected mortality (%)	14.49	21.70	32.46	49.13	64.97	87.67	93.23
	χ^2^	1.21	2.92	3.26	2.70	1.82	0.83	1.00
	*P*	0.27	0.09	0.07	0.10	0.18	0.36	0.32
	Mean difference (%)	4.64						

The backcross ACB-AbRS×ACBAbR showed that under low Cry1Ac concentrations inheritance of resistance was not significantly different to the monofactorial model (0.05–2.50 µg/g, *P* > 0.05). However, as the concentration of the toxin was increased (5.00, 25.00, 50.00 µg/g), the data showed a significant level of difference to the monofactorial model (*P* > 0.05), synonymous with polygenic inheritance (Table 6). A similar trend was observed with the ACB-AcRS×BtS backcross to Cry1Ac, with no difference to the monofactorial model under low concentrations but significance differences under high concentrations, again consistent with polygenic inheritance.

**Table 6:** Indirect tests for deviation between observed and expected mortality for monogenic and additive polygenic models in ACB-AbR and ACB-AcR to Cry1Ac.

Strains		Concentration (µg/g)						
		0.05	0.25	0.50	2.50	5.00	25.00	50.00
ACB-AbRS×ACB-AbR	Actual mortality (%)	17.36	21.53	28.47	43.06	59.72	77.78	94.44
	Expected mortality (%)	19.30	24.10	28.82	41.15	51.22	69.79	83.16
	χ^2^	0.35	0.52	0.01	0.22	4.17	4.36	13.09
	*P*	0.56	0.47	0.93	0.64	0.04	0.04	0.00
	Mean difference (%)	4.93						
ACB-AcRS×ACB-BtS	Actual mortality (%)	20.83	40.28	49.31	72.22	83.33	90.97	95.14
	Expected mortality (%)	10.42	16.27	21.98	38.54	48.09	68.89	80.21
	χ^2^	1.52	0.93	1.09	7.72	4.78	6.67	5.78
	*P*	0.22	0.34	0.30	0.01	0.03	0.01	0.02
	Mean difference (%)	3.89						

## 3. Discussion

Two resistant colonies of Asian corn borer reared in the laboratory under long-term selection pressure with either Cry1Ab or Cry1Ac evolved moderate levels of resistance and cross-resistance to several Cry1 Bt toxins. When reciprocal crosses were performed between ACB-AbR or ACB-AcR and the susceptible colony ACB-BtS, the F_1_ offspring showed no significant differences, and the data were in agreement with autosomal inheritance of the resistance with no maternal effects. Autosomal inheritance has been reported for many other insects with resistance to *B. thuringiensis* Cry toxins, including *Plutella xylostella* [29,30], *Trichoplusia ni* [31], *O. nubilalis* to Dipel ES, Cry1Ab and Cry1F [32,33,34], *Culex quinquefasciatus* to Cry11Aa and Cry4A+Cry4B [35], *Helicoverpa armigera* to Cry1Ac and Cry2Ab [36,37] and pink bollworm to Cry1Ac [38].

Generally, Bt toxins natively expressed in *B. thuringiensis* are different (even if it is slightly) from the “same” toxins when expressed in genetically modified plants or in *E. coli*. The source of toxins could result in different effects on susceptibility in the selected strains. Liang *et al.* used the Cry1Ac protoxin to study the mode of inheritance of lab-selected *H. armigera*. Results from that study indicated that inheritance was incompletely recessive [39]. In contrast, further studies by Wu *et al.*, where lab-selected colonies were challenged with activated Cry1Ac, inheritance of Cry1Ac resistance was dominant in *H. armigera*, thus indicating that the source and activation state of the toxin needs to be considered to ensure validity of the bioassay [40].

In the present study the resistant colonies ACB-AbR and ACB-AbR were challenged with each of the following insecticidal Cry proteins: Cry1Ab, Cry1Ac, Cry1F, and Cry1Ie. Both colonies showed a high level of cross-resistance to Cry1Ab and Cry1Ac; the observed cross-resistance to Cry1F was much lower. However, these two resistant colonies did not show any resistance to the Cry1Ie toxin. This study presents the first report of such a phenomenon in Asian corn borer, but confirms events that are widely known for other insects, where many accounts of cross-resistance to different Bt toxins have been reported. Gould *et al.* [13] report that a Cry1Ac resistant *H. virescens* colony was seen to be highly cross-resistant to Cry1Ab and Cry1Fa and also moderately cross-resistant to Cry2Aa, while showing almost no resistance to Cry1Ca and Cry1Ba [13]. Similarly, *P. gossypiella* with selected resistance to the Cry1Ac protoxin demonstrated a high level of cross-resistance to both the Cry1Aa and Cry1Ab protoxins, but cross-resistance to Cry1Bb protoxin was only present at low levels [41]. Furthermore, this phenomenon has been reported in *O. nubilalis*, where cross-resistance patterns are comparable to those presented here with the Cry1Ab-selected population exhibiting high levels of cross-resistance to Cry1Ac, and with cross-resistance to other toxins being less prevalent [26,42]. These findings suggest that a similar secondary protoxin might cause cross-resistance. Binding studies have shown that when cross-resistance occurs the secondary toxin interacts with the same binding sites as the initial protoxin used to rear the resistant colony. When cross-resistance is not observed, the two toxins interact with different binding sites [43]. The interaction between toxin and receptor, and variation within the receptor molecule may be used to predict the potential cross-resistance between different protoxins.

The offspring from the backcross of the resistant colonies were tested with different Cry1 proteins and showed different resistance patterns. For the strains tested with reciprocal Bt toxins, the inheritance of Cry1Ab and Cry1Ac resistance was polyfactorial in both Cry1Ac- and Cry1Ab- selected populations. However, the response of the ACB-AcRS×ACB-BtS colony to Cry1Ab was consistent with the monofactorial model of inheritance, but inheritance in the ACB-AcRS×ACB-BtS colony to Cry1Ab was polyfactorial. For the Cry1Ac protein, the inheritance pattern became more complicated than with Cry1Ab. Both colonies (ACB-AbRS×ACB-AbR and ACB-AcRS×ACB-BtS) fitted the monofactorial model under low concentrations but under high concentrations of toxin inheritance patterns were polyfactorial for the trait. These differences could result from experimental error as a result of the inherent problems with the classical Mendelian approach [35]. However, the backcross data for the offspring reflected the distinct evolutionary path of each colony in response to selection pressure with the different Cry1 toxins. Under low concentrations of the toxin, the reduced selection pressure allows many susceptible insects to survive the bioassay, therefore the data of inheritance was based on the highest concentration. Previous studies show that inheritance of Bt resistance is divergent amongst different species [44], with data collected so far for Lepidoptera [41], Diptera [45] and Coleoptera [46]. The present study using single Cry toxins showed that inheritance was either monofactorial or polyfactorial depending on the toxin and/or its concentration. The mechanism of inheritance and the resulting cross-resistance will benefit from continuing research to further develop this important field and to mitigate the negative effects of cross-resistance on the longevity of these important insecticidal toxins.

## 4. Experimental Section

### 4.1. Insect Colonies

Three laboratory colonies of *O. furnacalis*, a Bt susceptible colony (ACB-BtS), a Cry1Ab resistant colony (ACB-AbR) and a Cry1Ac resistant colony (ACB-AcR) were used for these studies. The ACB Bt susceptible colony (ACB-BtS) was collected from corn fields in Shaanxi province located in the summer maize region of central China. In this area, Bt maize is not planted and Bt spraying is not utilized for insect control, thus the ACB have not previously been exposed to Bt Cry1. ACB-BtS was reared on artificial diet developed at the Institute of Plant Protection (IPP), Chinese Academy of Agricultural Sciences (CAAS), Beijing. Cry1Ab- and Cry1Ac- resistant colonies have been maintained under selection pressure with Cry1Ab or Cry1Ac protein incorporated into artificial diet, respectively [15].

### 4.2. Bt Toxins

Trypsin-activated Cry1Ab, Cry1Ac, and Cry1Fa toxins (98% pure protein, Envirologix. Portland, ME, USA) were used to select the resistant colonies and for bioassays. The Cry1Ie toxin used in the bioassays was expressed as a recombinant protein in *E. coli* [47], which was provided by Beijing Institute of Technology. The selected colonies ACB-AbR and ACB-AcR were initially exposed to Cry1Ab and Cry1Ac in the rearing diet (2.5 ng/g, toxin/diet). The toxin concentrations were steadily increased to 4 μg/g and 24 μg/g for ACB-AbR and for ACB-AbR and ACB-AcR, respectively, to maintain the selection pressure at a level that resulted in 40%–70% mortality per generation.

### 4.3. Diet Bioassays

Cry1Ab, Cry1Ac and Cry1Fa proteins were initially dissolved in 50 mM sodium carbonate buffer (pH = 10) before mixing at the desired concentration in the agar-free semi-artificial diet, which was then divided into individual cells of 48-well trays. Each bioassay included eight different Bt concentrations plus a water control; one neonate (2–12 h after hatching) was exposed to the diet surface of each well. Three replicates were performed for each treatment at each of the different concentrations. Assays were carried out at 27 ± 1 °C with a photoperiod of L:D = 16:8 h and 70%~80% relative humidity. Mortality and larval weight were recorded after 7 days.

### 4.4. Genetic Crosses

Reciprocal mass crosses were carried out between ACB-BtS, ACB-AbR, and ACB-AcR. Virgin males and females were prepared by isolating individual pupae in 50 mL centrifuge tubes; subsequently 200 males and 200 females (approx.) were combined into one mating cage. A similar strategy was used to initiate the backcrosses. The following crosses and backcrosses were performed, with the female parent listed first:

(1) ACB-AbR×ACB-BtS (ACB-AbRS);
(2) ACB-BtS×ACB-AbR (ACB-SAbR);
(3) ACB-AcR×ACB-BtS (ACB-AcRS);
(4) ACB-BtS×ACB-AcR (ACB-SAcR);
(5) (ACB-AbR×ACB-BtS)×ACB-AbR (ACB-AbRS×ACB-AbR);
(6) (ACB-AcR×ACB-BtS)×ACB-BtS (ACB-AcRS×ACB-BtS);
(7) ACB-AbR×ACB-AcR (ACB-AbcR);
(8) ACB-AcR×ACB-AbR (ACB-AcbR).

The backcross method was used to estimate the number of alleles affecting resistance [48]. With this method, the determination of monofactorial or polyfactorial inheritance was tested from dose-response lines on backcross offspring compared with that of F_1_ offspring and the parents. If resistance is typical of the monofactorial model, the resulting progeny from the heterozygotes F_1_ (RS) backcrossed to the resistant colony (RR) or the susceptible colony (SS), will consist of a 1:1 ratio of RS:RR or RS:SS genotypes.

### 4.5. Statistical Analysis

The 50% lethal concentration (LC_50_) and slope were estimated for each bioassay with probit analysis (PoloPlus, LeOra software, 1987). Concentration *versus* mortality responses of ACB-AcR, ACB-AbR, ACB-BtS, F_1_ and backcross progeny were plotted using the LC_50_ values, chi-squares and slopes estimated from Probit analysis (PoloPlus, LeOra software, 1987). Chi-square and slopes parameters were used to determine the reliability of the data. The LC_50_ values were considered significantly different if their 95% fiducial limits did not overlap [49]. For mortality calculations larvae that had not reached the first instar and weighed ≤ 0.1 mg were considered dead. Thus, the practical mortality was obtained using the equation: Practical mortality (%) =100 × (number of dead larvae + number of surviving larvae that had a body weight of < 0.1 mg per larva)/total number of insects assayed. Mortality was corrected for control mortality using the method of Abbott [50]. The LC_50_ and 95% fiducial limits (FL) were determined by PoloPlus. Resistance ratios (RR_50_) were determined from concurrent bioassay tests on ACB-BtS and the resistance colonies using the same toxins and concentrations, and were calculated by dividing the LC_50_ of the resistance colony by the LC_50_ of ACB-BtS.

The degree of dominance (*h*) of the resistance or cross-resistance trait at specific concentrations was calculated as *h* = (ω_RS_ − ω_SS_)/(ω_RR_ − ω_SS_), where ω_RS_ is the fitness of the heterozygous offspring, ω_SS_ is the fitness homozygous susceptible parent, ω_RR_ is the fitness homozygous resistant parent. The fitness of the resistant homozygous parent at any treatment concentration was assumed to be 1. The fitness of the susceptible parent and the heterozygous F_1_ was estimated from the survival rate of the larvae at a specific treatment concentration divided by the survival rate of the resistant parent at the same concentration. Using this formula, an *h* value of 0 indicates fully recessive inheritance; an *h* value of 1 indicates a fully dominant trait; and an *h* value of 0.5 represents a co-dominant trait. When 0 < *h* < 0.5, inheritance of the trait is partly recessive, whereas when 0.5 < *h* < 1, the trait is partly dominant [30].

The indirect tests for deviation between observed and expected mortality for monogenic and additive polygenic models in ACB-AbR or ACB-AcR to Cry1Ab or Cry1Ac. Expected mortality in the backcross can be calculated directly from experimental data. The χ^2^ values and *P*-value can be calculated, based on the expected mortality and the test numbers following the method of Tabashnik *et al.* [48]. If the *P*-value > 0.05, inheritance of resistance is considered to fit the monofactorial model, but if the *P*-value < 0.05, then resistance inheritance is consistent with the polygenic model.

## 5. Conclusions

The results of this study suggest that *O. furnacalis* could evolve significant levels of resistance when the larvae are consecutively exposed to Cry1Ab or Cry1Ac toxins. The highest level of cross-resistance was observed between the Cry1Ab and Cry1Ac toxins, whereas only intermediate levels of cross-resistance to Cry1F toxin occurred. In contrast, no cross-resistance to Cry1Ie was observed in larvae previously exposed to either Cry1Ab or Cry1Ac. Mendelian backcross testing indicates that the inheritance of resistance to Cry1Ab or Cry1Ac toxins is either monofactorial or polyfactorial, depending on both the particular Cry toxin and/or its concentration. The availability of multiple toxins that do not cause cross-resistance could improve resistance management strategies.
